# Towards Ultrahigh Performance Concrete Produced with Aluminum Oxide Nanofibers and Reduced Quantities of Silica Fume

**DOI:** 10.3390/nano10112291

**Published:** 2020-11-19

**Authors:** Scott Muzenski, Ismael Flores-Vivian, Behrouz Farahi, Konstantin Sobolev

**Affiliations:** 1 Department of Civil and Environmental Engineering, University of Wisconsin-Milwaukee, Milwaukee, WI 53211, USA; scott.muzenski.ctr@dot.gov (S.M.); bfarahi@uwm.edu (B.F.); 2 Facultad de Ingeniería Civil, Universidad Autónoma de Nuevo León, San Nicolás de los Garza C.P. 66450, Mexico; ismael.floresvvn@uanl.edu.mx

**Keywords:** nanoalumina, ultrahigh performance concrete, high strength, cementitious composite, high-density polyethylene fibers, fiber-reinforced composites

## Abstract

Ultrahigh performance concrete (UHPC), which is characterized by dense microstructure and strain hardening behavior, provides exceptional durability and a new level of structural response to modern structures. However, the design of the UHPC matrix often requires the use of high quantities of supplementary cementitious materials, such as silica fume, which can significantly increase the cost and elevate the production expenses associated with silica fume handling. This paper demonstrates that a fiber-reinforced composite with properties similar to conventional UHPC can be realized with very low quantities of silica fume, such as 1% by mass of cementitious materials. The proposed UHPC is based on reference Type I cement or Type V Portland cement with very low C_3_A (<1%) that also complies with Class H oil well cement specification, silica fume, small quantities of Al_2_O_3_ nanofibers, and high-density polyethylene or polyvinyl alcohol macro fibers. Previous research has demonstrated that nanofibers act as a seeding agent to promote the formation of compact and nanoreinforced calcium silicate hydrate (C-S-H) clusters within the interparticle and nanofiber spaces, providing a nanoreinforcing effect. This approach produces a denser and stronger matrix. This research expands upon this principle by adding synthetic fibers to ultrahigh strength cement-based composites to form a material with properties approaching that of UHPC. It is indicated that the developed material provides improved strain hardening and compressive strength at the level of 160 MPa.

## 1. Introduction

**Figure 1:**
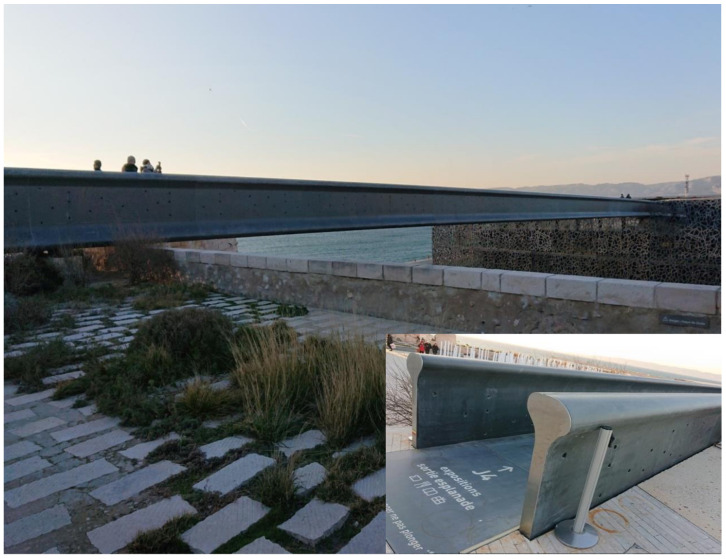
Application of ultrahigh performance concrete (UHPC) in MuCEM pedestrian bridge (Marseille, France).

To reduce maintenance and repair, which are frequently required for conventional structures such as bridges and sewer pipes, critical elements of modern infrastructure require the attainment of very high durability levels [1,2,3,4,5]. In addition, to minimize the threat to key structures and components in critical facilities, such as power plants, enhancing the durability of such elements is extremely important [6]. Ultrahigh performance concrete (UHPC) has emerged as a viable candidate for such critical elements because of a strain hardening response and low porosity, leading to improved strength, ductility, and durability. UHPC has been commercially available for a few decades, and ongoing research efforts are in place to determine the mechanical and durability response of new, more effective UHPC formulations. Commonly, UHPC has been used for prefabricated bridge elements, connections between the prefabricated bridge elements, and bridge overlays, among others (Figure 1) [7,8,9]. UHPC is also used for soil stabilization and bridge foundations [10,11,12,13]. The application of nanotechnology in concrete is another emerging development, transforming modern infrastructure [14,15]. The use of nanotechnological developments, and specifically nanoparticles, towards the engineering of an ultrahigh performance cement-based matrix may prove to be beneficial to reduce the quantities of silica fume (SF) commonly used to obtain high strength UHPC (commonly up to 25% of cementitious materials by mass).

Although there are no standard definitions and varying interpretations of UHPC, the most commonly used definition states that the material must have a water to cementitious material (W/CM) ratio less than 0.25, a compressive strength of at least 150 MPa, sustained pre- and post-cracking tensile stress of at least 5 MPa, and a very dense microstructure as required for improved durability [16,17,18,19]. However, various cement-based composites with properties slightly deviating from this specification may still be considered as an attractive alternative, providing effective use for the intended application. In fact, some authorities in Switzerland, Canada, and France have included fiber-reinforced cement-based composites (FRC) with compressive strengths as low as 120 or 130 MPa in UHPC-class materials [20,21,22].

The type of binder and the type of supplementary cementitious material (e.g., silica fume and metakaolin (MK)) are of crucial importance for UHPC performance. Silica fume is often considered to be a critical material to create UHPC with superior strength [23,24]. Silica fume is an ultrafine pozzolanic supplementary cementitious material with smaller average particle diameter and larger specific surface area than other cementitious materials. These characteristics allow SF to pack the void space and to improve the interfacial transition zone between the aggregates and larger cement particles [24]. This, in turn, results in a very dense cementitious matrix, which is often a limiting factor in the conventional material’s strength. In UHPC, silica fume is often considered at an optimal dosage of up to 25% [14,25]; however, the use of higher quantities has proven acceptable.

The use of high quantities of silica fume may prove to not be economical due to its limited supply and cost. Besides, the incorporation of large volumes (up to 25%) of SF represents a technical challenge. Here, the nanoparticles are also an expensive additive; however, these have proven to be effective at very low quantities (less than 1%) [26]. The combination of submicron-sized silica fume with nanoparticles or nanofibers may be beneficial by providing a better packing degree and thus an even denser cementitious matrix. This development may be accomplished by replacing a large portion of silica fume with a small quantity of well-dispersed nanoparticles [27]. Nanosilica is one of the most common nanomaterials currently used in concrete. It has been observed that the use of nanosilica in cementitious materials can generate denser packing of hydration products, refinement of the pore structure, and improved interfacial transition zone [28,29,30,31]. The use of this type of nanomaterial accelerates the hydration of cementitious products by acting as a seed for the nucleation of calcium silicate hydrates (C-S-H) [32]. Al_2_O_3_ nanofibers may even act as a nucleation site for the formation of C-S-H [14]. The nanofibers have the potential to provide some reinforcing effect to the C-S-H globules. The use of nanofibers also results in significant improvement of compressive strength and increased density in cement-based composites [26]. These properties make Al_2_O_3_ nanofibers a great candidate for use in UHPC.

Another critical component of UHPC is fiber reinforcement. While various types of fibers are used in FRC, UHPC typically utilizes steel fibers. The properties of fibers can have a significant effect on the performance of the composite. Polyvinyl alcohol (PVA) fiber is a popular reinforcing material that is extensively used in engineered cementitious composites (ECC) and composites designed for strain hardening response. The ECC is known for its exceptional ductility and strain hardening behavior [33]. High-density polyethylene (HDPE) fiber is another type of reinforcement that has demonstrated contribution to exceptional multi-cracking behavior and excellent load transferring ability after crack formation, resulting in a strain hardening response. In FRC with strain hardening, the loads after first crack are restrained by controlled crack openings and continue to increase until the fibers begin to rupture [34]. Usually, the aforementioned fibers have a high aspect ratio, which allows superior performance [23,25,26,27,28,29,30,31,32,33]. Additionally, there are numerous other forms of fiber reinforcement that are effectively used in cement-based composites.

The preliminary work on the incorporation of Al_2_O_3_ nanofibers into a high strength cementitious matrix has demonstrated encouraging performance [26]. In this work, it was hypothesized that combining nanoengineered matrices with high strength and high aspect ratio discontinuous fiber reinforcement might result in an advanced material with properties meeting or exceeding the specifications for ultrahigh performance concrete. The objective of this research was to study this hypothesis in two parts:

- First, the effect of nano-Al_2_O_3_ fibers with small amounts of supplementary cementitious composites, such as silica fume or metakaolin, in mortars was examined. This was done with both Type I and Type V Portland cement systems. This helped to determine if nano-Al_2_O_3_ fibers could be used with Type I Portland cement to form potential UHPC.
- Second, using data from the first part of this research along with data from previous high strength cementitious matrices with Al_2_O_3_ nanofibers [26], ultrahigh performance concrete was created by adding synthetic fibers.

## 2. Experimental Procedures

### 2.1. Materials

The cementitious materials used in this research included ASTM C109 [35]. Type I portland cement, two Type V portland cement products (also conforming to Class H oil well cement specification), metakaolin, and silica fume. The chemical composition of cement is reported in Table 1 using an X-ray fluorescence (XRF) technique. The Type V portland cement products had a very similar chemical and mineral composition.

The typical morphology of silica fume was represented by almost perfect spherical particles between 0.2 and 1 µm in diameter. On the contrary, metakaolin was characterized by rough particles with a size range between 0.8 and 12 µm.

**Figure 2:**
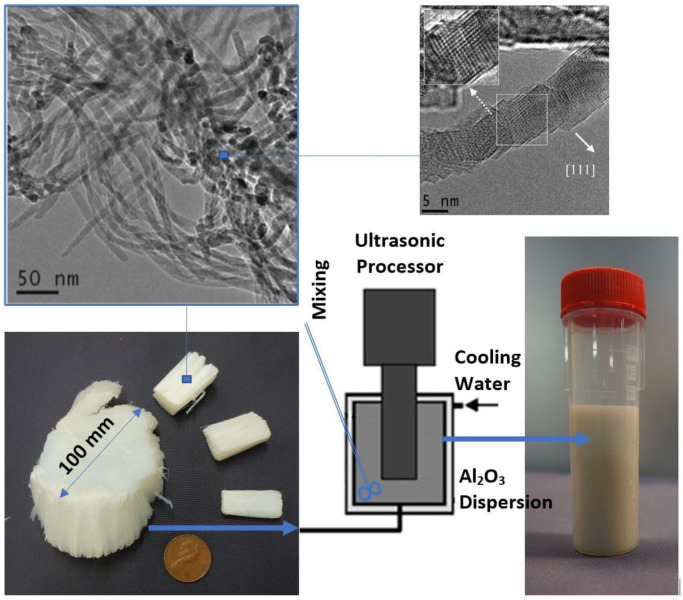
The dispersion of Al_2_O_3_ Nanofibers: Top—Nanofibers of Al_2_O_3_ (left) under SEM and TEM (right, courtesy of AFN Technologies); Bottom—The schematics of nano-Al_2_O_3_ preparation from disc as received into dispersion using ultrasonic processor (left to right).

The high-range water reducing admixture used in the research was a commercially available polycarboxylate superplasticizer (PCE-SP) with 31% concentration of solids. The nano-Al_2_O_3_ fibers used were chemically pure crystalline alumina with a surface area of 155 m^2^/g. The single crystal tensile strength of the fiber was 12 GPa, while the modulus was 400 GPa. The typical fiber diameter was 10–20 nm, and it was delivered in disks with fiber lengths of 50 mm (Figure 2). The fibers were synthesized from an aluminum melt and then grown to the aforementioned lengths. Upon dispersion through ultrasonication [26], the fibers maintained their diameter but broke down to lengths between 10 and 65 µm. Due to potential health hazards and dangers of nanoparticles becoming airborne, special precautions were taken while the nanofibers were handled. To comply with the safety protocols, nanomaterials were weighed and added to water in a glove box to assure no airborne particles. The lab-made suspension of nano-Al_2_O_3_ with superplasticizer was used for the preparation of fiber-reinforced UHPC, and as-manufactured (predispersed) nano-Al_2_O_3_ fiber solution was used for the study of hydration effects and performance evaluation of UHPC matrices. These predispersed nano-Al_2_O_3_ fibers were found to contain some quantity of a surfactant.

PVA fibers and HDPE fibers were used in this research as reinforcement. The PVA fibers had a length of 8 mm, thickness of 15 dtex, diameter of 40 µm, Young’s Modulus of 40 GPa, and tensile strength of 1.6 GPa. The HDPE fibers had a density of 970 kg/m^3^, length of 12 mm, diameter between 12 and 21 µm, axial tensile strength of 3.6 GPa, and axial tensile modulus of 116 GPa.

Standard-graded silica sand conforming to ASTM C778 [36] was used in this research. The sand was graded, so 96% of the aggregates fell between the No. 30 and No. 100 sieves.

### 2.2. Experimental Design

The experimental program was divided into two main parts. The first part was to compare the effect of cement type and reactive silica type on the performance of UHPC matrixes. The cementitious materials used in this part contained Type I Portland cement, Type V-A cement, SF, and MK. The W/CM ratio for the samples was 0.3 for Type I cement and 0.225 for Type V-A cement. The investigated samples had sand to cementitious materials (S/CM) ratio of 1.0 and contained high-range water-reducing admixture (SP) and predispersed (by the manufacturer) nano-Al_2_O_3_ fibers at a dosage of 0.15% and 0.25% (as solid content by mass of cementitious materials), respectively. The approach for effective utilization of nanomaterials at very small dosages, less than 1%, has been previously discussed [26,37]. The idea of nanoparticle combination with reduced quantities of micron and submicron-sized powders (such as silica fume and metakaolin) was proposed [15] and explored in [26].

Earlier work has demonstrated that the use of Al_2_O_3_ nanofibers and Class H cement-based matrices can significantly improve the compressive strength [26]; however, it has yet to be determined if the nanofibers can have a similar effect on the performance of fiber-reinforced composites. Therefore, by first testing similar materials in mortars prior to determine if the required compressive strengths for UHPC could be obtained in Type I portland cement systems, a reduced fiber-reinforced composite matrix could be performed.

An additional aspect that was considered was the S/CM ratio. In the first part of the research program, a S/CM ratio of 1.0 was used to see if a cement-based material with a relatively high S/CM ratio could be used to form UHPC. As shown in the Mechanical Properties section later in this paper, the compressive strengths of the mortars barely reached the 150 MPa threshold. It was determined that when fibers were to be added in the second part of the research program, the higher S/CM ratio would not provide enough bonding of the fibers to the cementitious matrix and the mechanical properties, including the compressive strength, might be reduced. For this reason, the S/CM ratio was reduced to 0.5 in the second portion of the research program.

The W/CM ratio was varied between Type I and Type V portland cement in order to maintain similar flow and consistency. The W/CM ratio was even further reduced in the second part of the research program because the lower quantity of sand demanded less water. Here, the lower flow might be attributed to the fibers, whereas the consistency was visually observed to be similar.

In the second part of the research program, the experimental program was designed based on the previous work to determine the mechanical properties of composites containing Al_2_O_3_ nanofibers dispersed in the lab, along with different types of macroreinforcing fibers. Two different types of fibers (PVA and HDPE) were considered and used at quantities of 2% by volume. The nanofibers were dispersed in the lab and used at a dosage of 0.5% by mass of the cementitious material. Although the lab-dispersed nanofibers were used in this portion of the study, it is expected that a manufactured predispersed nanofiber would be used in most applications. This would result in a time-saving measure for the production of the composite. The Type V Portland cement used in this portion of the research program was intended to be the same as the material used in the first part of this research program. However, due to availability, material from the same blending was not available. Despite this, the chemical composition, as can be seen in Table 1, was almost identical, therefore reducing any uncertainties. All samples used silica fume as a 1% replacement of cement. It was found that such a combination with Al_2_O_3_ nanofibers could provide a compressive strength similar to the composites with significantly higher quantities of silica fume [26]. These samples used W/CM ratio of 0.173, S/CM ratio of 0.5, and high-range water reducing admixture (SP) at a dosage of 0.10 (by solid content) by mass of cementitious materials. A complete experimental matrix of the reported research is summarized in Table 2.

### 2.3. Preparation of Cement-Based Composites

#### 2.3.1. Dispersion of Al_2_O_3_ Nanofibers

To prepare the dispersion of nanofibers for the second part of the research program, a full tablet of Al_2_O_3_ nanofibers (Figure 2, typically between 35 and 45 g) was placed in a container. Deionized water and PCE-SP were added to the container and hand mixed briefly using a stirring rod to break up any large agglomerates. The resulting slurry consisted of 94.6% deionized water, 3.8% nanoalumina fibers, and 1.6% PCE-SP (solid material) by weight. A high-speed mixer (HSM) at 8000 rpm in combination with ultrasound processing at 20 kHz and an amplitude of 85% (21.5 µm) was used to disperse the slurry. Cold water and ice were used on the exterior of the container to keep the dispersion below 50 °C. The water and ice were replaced regularly throughout the dispersion process. The slurry was then left to disperse for 3 h.

#### 2.3.2. Cement-Based Composites

The composites were mixed according to ASTM C305 Standard Practice for Mechanical Mixing of Hydraulic Cement Pastes and Mortars of Plastic Consistency [38] with some modifications for the addition of fibers. Once the mortar had been mixed in accordance with the standard, the fibers were slowly (within 30–60 s) added while mixing at medium speed (198 rpm) and then mixed for an additional 90 s. A portion of the fresh composite was tested for flow and then placed back into the mix for additional mixing at medium speed for 30 s.

The composite was placed into 50.8 mm × 50.8 mm × 50.8 mm cube molds for compressive testing and 14 mm × 40 mm × 160 mm beam molds for four-point flexural and tension testing. Prior to casting the fresh composite, each mold was sprayed with a release agent for ease of demolding after 24 h. Beam or cube molds were filled with cement-based composites and cast in two layers, each being compacted using a standard hard rubber tamper (13 mm × 25 mm × 152 mm) and leveled before placed in the curing chamber. The cube molds were compacted in accordance with ASTM C109 [35], while the beam molds were compacted with a total of 40 tamps (20 tamps on each layer).

After placement of the composites in the molds, the molds were covered with glass plates and placed in a curing room at 20 ± 3 °C and relative humidity of no less than 90% as per ASTM C192 standards [39]. The specimens were then removed from the molds after 24 h. One-day tests were then performed on the appropriate specimens, and the remaining specimens were placed in a lime water bath until the testing age.

### 2.4. Evaluation of Cement-Based Composites

The flow of fresh composites was tested using a 254 mm (10 inch) flow table as per the ASTM C230 standard [40]. To quantify the effect of nano-Al_2_O_3_ fibers on the hydration process, the heat evolution rate and total hydration heat were determined using mortar samples in accordance with ASTM C1679 [41] by an isothermal calorimeter (TAM Air from TA Instruments) at 25 ± 1 °C for 72 h.

Compressive strength tests were performed in accordance with ASTM C109 [35]. These specimens were tested with an automatic compression machine and loaded at a rate of 1.4 kN/s. Four-point flexural testing was performed to determine flexural behavior. The end supports were spaced 120 mm apart with the middle loading supports spaced 40 mm apart, which created a third point bending test. The beams were then loaded at a rate of 1.2 mm/min to observe the load–deflection (stress–strain) behavior after the initial cracking. The deflection at the top supports was recorded from the testing frame and was used to interpolate the deflection at the midspan of the beam and ultimately calculate the flexural strain of the composite using ASTM D7264 [42]. The direct tension tests were performed on samples of the same 160 mm long × 14 mm tall × 40 mm wide beams. These samples were then cut to form a dog-bone shape with a width of 34.5 mm and a gauge length of 76.2 mm to ensure that failure did not occur near the supports. The samples were placed into the testing frame and loaded at a rate of 0.2 mm/min until failure (or when a significant reduction in load-carrying ability was observed).

## 3. Results and Discussion

### 3.1. Fresh Properties

Mortar flow test demonstrated that compositions with nano-Al_2_O_3_ fibers based on Type I and Type V-A cement had self-consolidating properties with a flow of more than 110% as reported in Table 2. Only the reference composition based on Type I cement had a flow of 98%.

The performance of UHPC was relatively consistent among all the compositions. Although not tested, the viscosity of the samples with nanofiber dispersion appeared to be lower. This may be attributed to the incorporation of PCE surfactants used for the dispersion of the nanofibers [14,15]. Here, it is unclear if any amount of surfactant that was used to aid in the dispersion of the nanoparticles may still be actively contributing to the workability of the fresh mixture. It may be envisioned that during the dispersion process, the surfactant is adsorbed and so consumed to suspend the nanofibers [26]. Therefore, the system was engineered to avoid any stiffening effect when used in cement-based materials. This design approach is based on the results of previous studies [26,37].

### 3.2. Heat of Hydration

**Figure 3:**
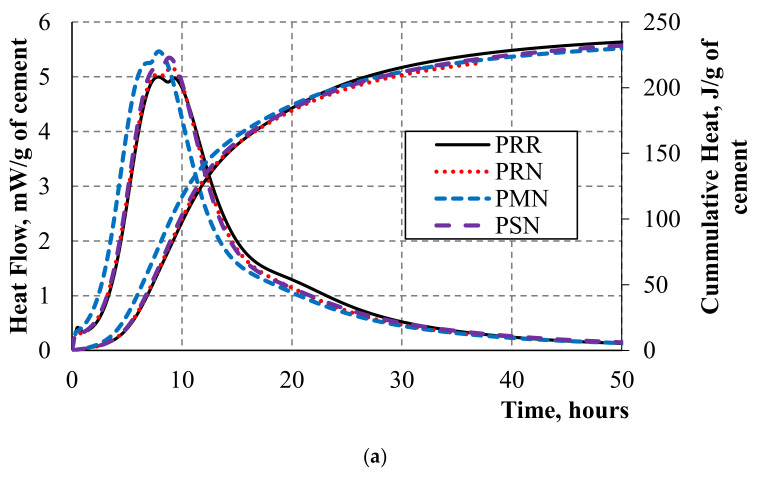

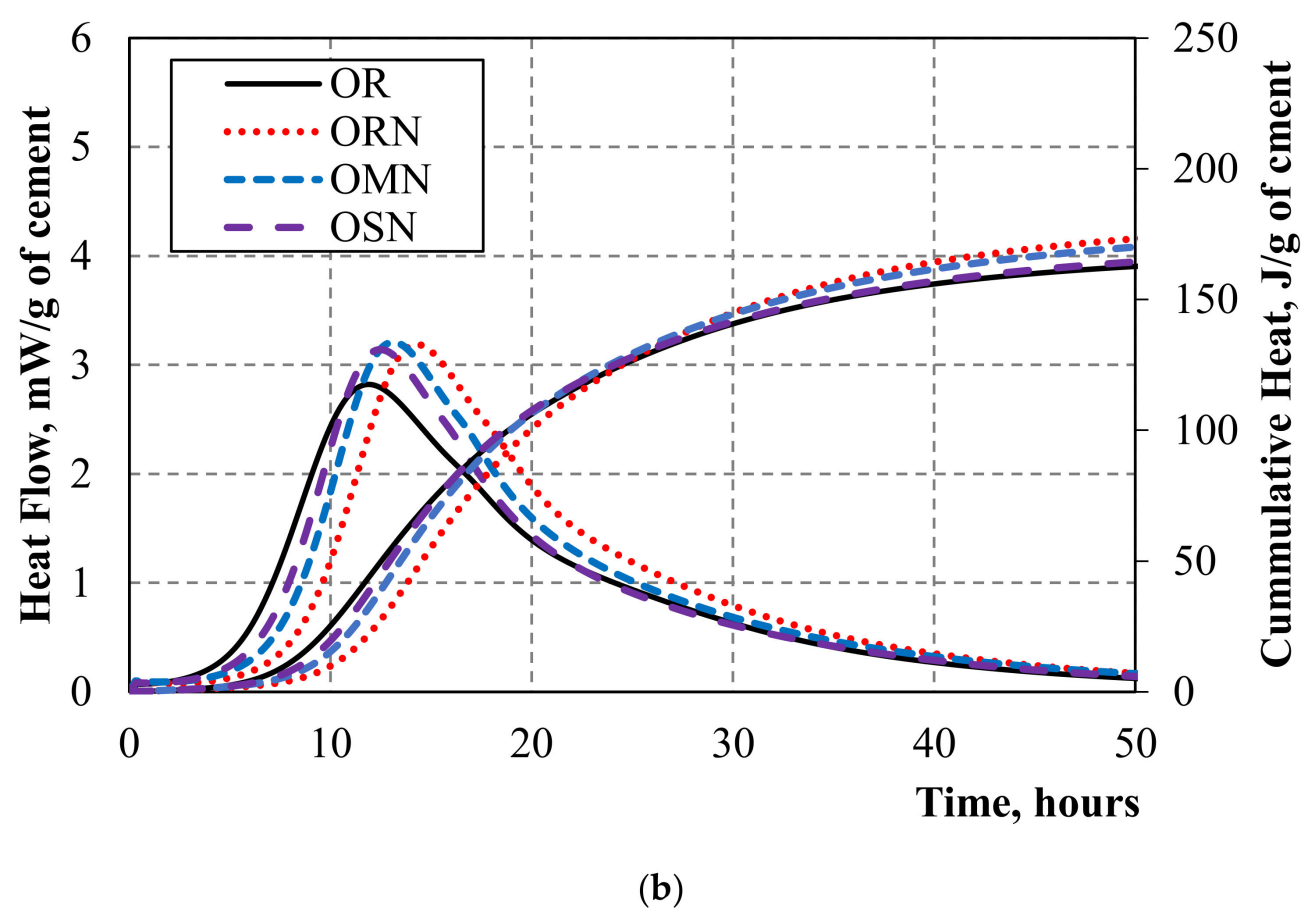
Heat of hydration of mortars with (**a**) Type I Portland cement and (**b**) Type V cement.

Figure 3 reports on the heat flow and total hydration heat of investigated mortars with nano-Al_2_O_3_ fibers. The obtained results demonstrate that replacement of cement with small quantities of nano-Al_2_O_3_ combined with SF or MK can lead to the acceleration of hydration. It was observed that the compositions with metakaolin had faster hydration than those containing silica fume. Here, the aluminate phases in metakaolin can accelerate the hydration process. The use of nanofibers in compositions with portland cement did not affect the hydration process significantly [43,44,45]; on the other hand, the addition of Al_2_O_3_ nanofibers led to a delay of hydration in Type V cement-based compositions. This delay cannot be attributed to the chemical properties of Al_2_O_3_ as the increased surface area of nanoparticles should typically result in accelerated hydration. However, the dispersion of nano-Al_2_O_3_ fibers was realized with the help of surfactants (which are commonly very similar to PCE superplasticizers used in concrete technology), so the delay may be explained by the combined effect and interaction of surfactants and PCE, as was previously discussed in [14]. The use of nanofibers in compositions with Type V cement resulted in an increased peak heat flow in comparison with the reference mix. Furthermore, it was observed that the compositions with Portland cement had a higher peak heat flow, and the total released heat was higher when compared to the systems based on Type V cement. The reason for this response is that the Type V cement had a coarser grain size and also had a reduced content of C_3_S and a very low content of C_3_A.

### 3.3. Mechanical Properties

**Figure 4:**
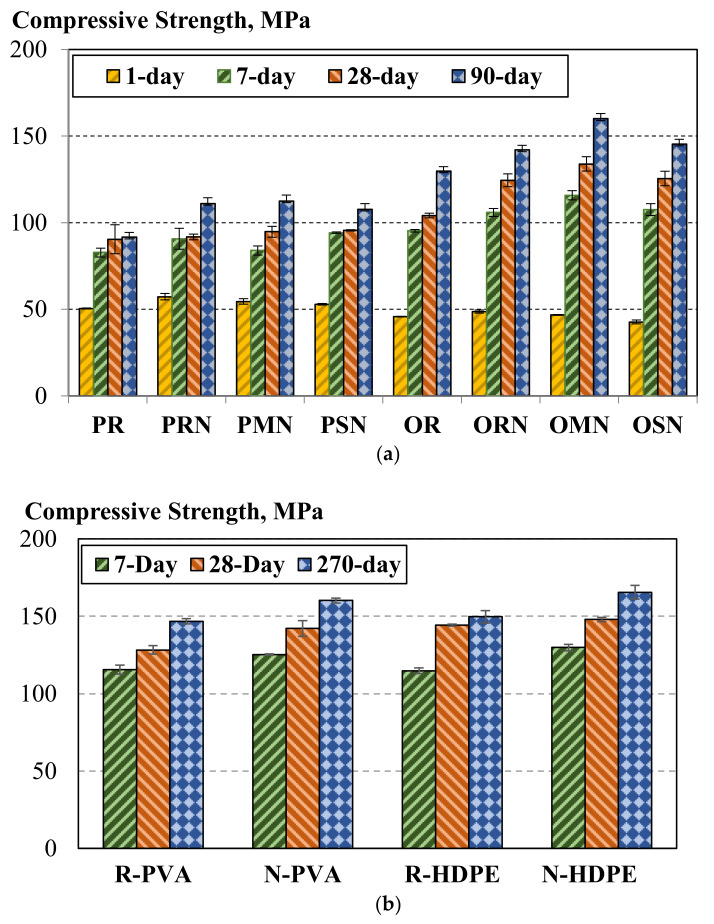
Compressive strength of (**a**) investigated mortars and (**b**) developed fiber-reinforced composites.

It was observed that the addition of Al_2_O_3_ nanofibers led to an increase in the compressive strength of the tested mortars at 90-day age, for example, for Portland cement-based compositions, a 21% increase vs. the reference without nanofibers or supplementary cementitious materials was achieved (Figure 4a). It was evident that the use of small quantities of SF and MK (1% cement substitution) improved the compressive strength of mortars with nanofibers. The aluminate phase of metakaolin resulted in acceleration of the hydration process, which, in turn, led to higher early-age strength attainment compared to the compositions with silica fume. It was observed that the early-age strength of compositions based on Type V cement was lower than that observed for Portland cement. These results are well correlated to the heat of hydration study. At later ages, the Type V compositions demonstrated better performance in terms of compressive strength. The best performance was for the Type V composition with metakaolin and nanofibers with a compressive strength of 160 MPa at 90-day age, meeting the criteria required for ultrahigh performance concrete.

The difference in compressive strength between samples with Type I and Type V portland cement systems demonstrates that in its current formulation, Type I portland cement systems with small amounts of supplementary cementitious and Al_2_O_3_ nanofibers may not provide adequate strengths to produce UHPC. This was a critical factor in not including Type I Portland cement systems in the fiber-reinforced portion of this research. Furthermore, combining data from previous research [19] with that of this research using Type V cement, a reduced experimental matrix using fiber-reinforced composites was achievable in the second part of this research program.

The compressive strength data demonstrated that the use of Al_2_O_3_ nanofibers in fiber-reinforced composites resulted in the attainment of a compressive strength exceeding 150 MPa (Figure 4b). The reported 28-day compressive strength was slightly lower than the 150 MPa threshold for UHPC, while the 270-day results were even further improved. The extended time for the material to reach the threshold strength may be attributed to the physical and chemical properties of Type V cement. The use of Al_2_O_3_ nanofibers resulted in a 9% and 11% increase in compressive strength for the samples with PVA and HDPE fibers, respectively. This feature further promotes the concept of using small dosages of Al_2_O_3_ nanofibers to produce the matrices for ultrahigh strength composites. The effective performance may be attributed to the seeding effect of the nanofibers promoting the nucleation of C-S-H. Additionally, these nanofibers can add a reinforcing effect to C-S-H globules, mitigating shrinkage and delaying the transformation of micro- and sub-microcracks into macrocracks [26]. The type of fiber (PVA or HDPE) did not appear to have any considerable effect on the compressive strength (as observed deviation can be explained by the variations in entrapped air content). This response was expected as the fibers typically would not provide any benefit to the composite loaded in compression. The only scenario where the fiber type may reduce the compressive strength is if the fibers were to lower the workability and thus result in an increased volume of entrapped voids.

**Figure 5:**
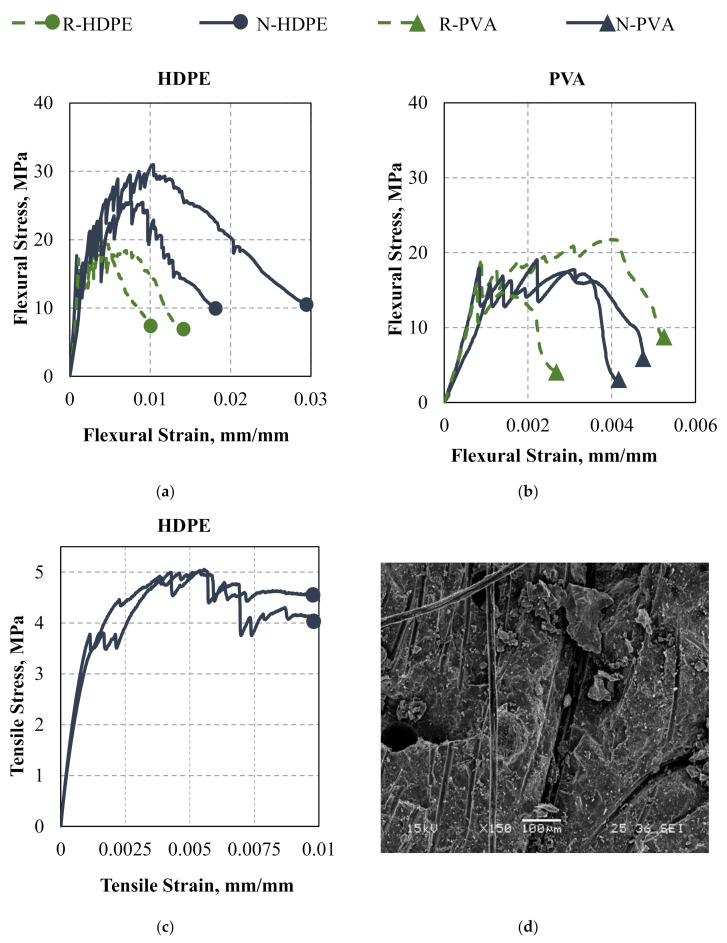
The 28-day performance of developed fiber-reinforced composites: flexural behavior of HDPE composites (**a**) and PVA composites (**b**); tensile response of Al_2_O_3_ HDPE fiber (N-HDPE) composites (**c**) and fractured Al_2_O_3_ HDPE fiber composite under SEM (**d**).

The flexural and tensile tests of fiber-reinforced composites demonstrated the improved strain hardening performance of samples with high-density polyethylene fibers (Figure 5). All samples appeared to have a similar elastic and first crack response (which, to no small extent, is controlled by the matrix); however, the post-cracking performance differed among all tested matrices and fibers used. Here, the samples with HDPE fibers outperformed the composites based on polyvinyl alcohol fibers by resisting high flexural stresses and providing better ductility (Figure 5a,b). Note that the flexural strain scale differs between Figure 5a,b. The HDPE fibers had a higher tensile strength, higher modulus, and higher aspect ratio, which is the main reason for the improved flexural performance. As expected, the HDPE series with Al_2_O_3_ nanofibers performed better than the control composites. Based on the compressive strength data, the addition of nanofibers produced a stronger matrix and a better grip to the fibers. It may be expected that the higher strength would result in a more brittle response; however, this was not the case as the ductility of the samples with nanofibers was improved (potentially, this was due to nanoscale reinforcement). The use of high-density polyethylene fibers of smaller diameter, combined with the seeding effect produced by the Al_2_O_3_ nanofibers, resulted in a denser matrix, especially near the fiber interface (Figure 5d). This phenomenon produced exceptional strain hardening behavior by allowing the fibers to have a better response and controlled fiber pull-out from the cementitious matrix. Better composite action and the ability of the HDPE fibers to provide an ultimate reinforcing effect in flexure are the main features of advanced composites. Furthermore, it may be hypothesized that the nanofibers would provide some reinforcing effect to the C-S-H formations [26]. This would stiffen the matrix and, theoretically, bridge the cracks at the C-S-H level, resulting in an improved flexural response.

Although flexural behavior is not often used to classify ultrahigh performance concrete, it may provide some indication of its ductility. High flexural stress and sustained strain hardening behavior of the samples with both Al_2_O_3_ nanofibers and HDPE fibers indicate that the composite exhibits the properties required for UHPC. Figure 5c demonstrates the 28-day tensile performance of the samples with both Al_2_O_3_ nanofibers and HDPE fibers under direct tension. It was reported that the failure of the samples occurred within the gage length by localization of one of the multiple cracks formed upon loading. This is represented on the stress–strain curve as the peak stress or the point where the tensile stress begins to continuously decrease.

Samples without nanofibers were not tested for direct tension. Additionally, samples with PVA fibers were not tested for direct tension. As these samples had significantly lower flexural behavior, it was assumed that they would have significantly lower tensile behavior; thus, they were not tested.

The N-HDPE samples provided tensile results approaching those of UHPC. Typically, ultrahigh performance concrete requires the first crack and sustained tensile stress of at least 5 MPa. Here, the maximum tensile stress was 5 MPa; however, the first crack stress and sustained stresses were slightly lower than set by this threshold. The observed tensile strains at the point where the maximum tensile stress was observed was in the range of 0.005 to 0.006 mm/mm. These values are consistent if not higher than those observed in conventional UHPC. If tested at later ages, the tensile stresses may have exceeded the required threshold. This data demonstrates that it may be possible to create a strain hardening UHPC with bilinear tension properties using a small amount of silica fume and nonmetallic fibers, whereas conventional UHPC has high quantities of silica fume or metakaolin and requires steel fibers to produce strain hardening behavior.

### 3.4. Towards Ultrahigh Performance Benchmarks

The cement-based materials containing nano-Al_2_O_3_ fibers, HDPE fibers, and only 1% of silica fume at 28-day age, provided properties approaching the benchmarks set for ultrahigh performance concrete. The compressive strength results of the developed composites provided the required strength benchmarks to be considered as ultrahigh performance at a 90- or 270-day age. Still, the seven-day age strength of 100 MPa may be attractive for many applications. The 28-day tensile behavior had similarly impressive characteristics, with properties just slightly under the requirements for ultrahigh performance concrete. In order to improve the tensile performance, some small modifications could be made to the mix design to optimize the packing and decrease the porosity of the matrix. Adding some additional volumes of silica fume would help to achieve this objective [30]; however, the motivation of this research was to create ultrahigh performance concrete with considerably reduced quantities of silica fume. The best strategy to achieve this goal would be by optimizing the combined distribution of particles used for the composite. As sand particles (diameter of 150–600 µm), HDPE fibers (diameter of 12–21 µm), Type V (Class H) cement (1.0–150 μm), silica fume (diameter of 0.2–1.0 µm), and Al_2_O_3_ nanofibers (diameter of 0.01–0.02 µm) were used in the current mix, the gaps between the types of particles can still be present, so further optimization can be beneficial. These gaps may be further filled by replacing either some volumes of cement or sand with another supplementary cementitious material, such as finely ground granulated blast furnace slag or fly ash [45]. The combination of nano-Al_2_O_3_ fibers with nano-SiO_2_ particles could be another option [29,37]. This development should result in a denser matrix and thus further increase compressive strength and improve tensile and flexural behavior.

## 4. Conclusions

This paper demonstrated the feasibility of adding synthetic fibers to an ultrahigh strength cement-based composite with considerably reduced quantities of silica fume to generate properties approaching ultrahigh performance concrete. This objective was achieved by using Al_2_O_3_ nanofibers, which proved to be an effective means to improve the compressive strength and flexural performance of fiber-reinforced composites at a 90-day age. Based on previous work [14], it was theorized that the nano-Al_2_O_3_ fibers are acting as the seeds promoting the nucleation of calcium silicate hydrates, therefore resulting in a denser microstructure characterized by filled-up void space between the hydrating cement particles. Otherwise, this space would have to be filled with higher quantities of silica fume. The use of ultrahigh performance concrete in engineering applications is becoming more desirable because of its high strength and high durability. However, the high costs associated with UHPC technology may sometimes act as a deterrent. By replacing high volumes of silica fume in UHPC with small quantities of Al_2_O_3_ nanofibers, a more economical and therefore more effective UHPC material can be produced. The reported data for the cement-based composite based on Type V (Class H) cement, Al_2_O_3_ nanofibers, small quantities of silica fume, and high-density polyethylene fibers prove that the developed material is approaching the benchmark properties set for UHPC. Similar compositions with Type I portland cement systems may not provide the adequate compressive strength to be considered UHPC. Future work would require the fine-tuning of the cementitious matrix to result in properties exceeding the requirements for UHPC with regard to tensile strength. The performance enhancement of the developed composite can be further realized in systems with finely ground granulated blast furnace slag, fly ash, and nano-SiO_2_. The role of the surfactant in the dispersion of Al_2_O_3_ would also have to be addressed in future work.

Due to a very low W/C ratio, low permeability, and very high density of the matrix, the durability of developed UHPC is assumed to be exceptional; however, experimental verification of durability parameters, including new testing methods for UHPC, may still be necessary and so needs to be addressed in further research.

## Figures and Tables

**Table 1:** Chemical composition of cement based on X-ray fluorescence (XRF) analysis.

Component	Type I	Type V-A	Type V-H
SiO_2_, %	19.6	21.4	21.8
Al_2_O_3_, %	4.6	3.0	3.1
Fe_2_O_3_, %	3.0	4.5	4.5
CaO, %	64.2	64.4	64.3
MgO, %	2.5	2.9	2.7
SO_3_, %	2.7	2.8	1.6
Na_2_O, %	0.2	0.1	0.2
K_2_O, %	0.5	0.2	0.2
C_3_S, %	69.5	65.0	64.3
C_2_S, %	3.8	12.3	14.0
C_3_A, %	7.1	0.3	0.6
C_4_AF, %	9.1	13.7	13.7

**Table 2:** Experimental matrix for fiber-reinforced cement-based composites (FRC) with Al_2_O_3_ nanofibers.

Sample ID	Cement Type	W/CM Ratio	S/CM Ratio	SP, % of CM	MK, % of CM	SF, % of CM	Nano-Al_2_O_3_, % of CM	% Fiber Volume	Flow, %
PR	Type I	0.3	1	0.15	0	0	0	-	98
PRN	Type I	0.3	1	0.15	0	0	0.25 *	-	>110
PMN	Type I	0.3	1	0.15	1	0	0.25 *	-	>110
PSN	Type I	0.3	1	0.15	0	1	0.25 *	-	>110
OR	Type V-A	0.225	1	0.15	0	0	0	-	>110
ORN	Type V-A	0.225	1	0.15	0	0	0.25 *	-	>110
OMN	Type V-A	0.225	1	0.15	1	0	0.25 *	-	>110
OSN	Type V-A	0.225	1	0.15	0	1	0.25 *	-	>110
R-PVA	Type V-H	0.173	0.5	0.1	0	1	0	2% PVA	57
N-PVA	Type V-H	0.173	0.5	0.1 **	0	1	0.5	2% PVA	56
R-HDPE	Type V-H	0.173	0.5	0.1	0	1	0	2% HDPE	44
N-HDPE	Type V-H	0.173	0.5	0.1 **	0	1	0.5	2% HDPE	53

* The predispersed nano-Al_2_O_3_ fibers were found to have some surfactant present. ** Superplasticizer (SP) at a content of 0.1% of cementitious material (CM) was added during the mixing process. Additional SP was present in the nano-Al_2_O_3_ dispersion, but it is unknown if this remained active after the nanodispersion.
